# Prevalence and impact of peer victimisation among youth seeking treatment at a tertiary psychiatric institution in Singapore: a cross-sectional study

**DOI:** 10.1186/s12991-022-00424-z

**Published:** 2022-11-24

**Authors:** Mythily Subramaniam, Shazana Shahwan, Edimansyah Abdin, Swapna Verma, Bhanu Gupta, Boon Yiang Chua, Yunjue Zhang, Rajeswari Sambasivam, Siow Ann Chong

**Affiliations:** 1 Research Division, Institute of Mental Health, Buangkok Green Medical Park, 10 Buangkok View, Singapore, 539747 Singapore; 2 Saw Swee Hock School of Public Health, National University of Singapore, Singapore, Singapore; 3 Medical Board, Institute of Mental Health, Singapore, Singapore; 4 South Region, Institute of Mental Health, Singapore, Singapore

**Keywords:** Peer victimization, Youth, Mental illness, Depression, Quality of life

## Abstract

**Background:**

Peer victimization is common among adolescents and leads to negative consequences. However, few studies have examined the extent of peer-victimization and its correlates among adolescent patients in a psychiatric setting. The current study aimed to examine the prevalence and correlates of peer victimisation among youth with mental illness and to examine its association with depressive symptoms and health-related quality of life (HRQOL).

**Methods:**

A sample of 239 youths aged 15–24 years were recruited from the outpatient clinics of a tertiary psychiatric hospital in Singapore using convenience sampling. All participants were administered the Multidimensional Peer Victimisation Scale (MPVS), Short Form 12 (SF-12) questionnaire and the Patient Health Questionnaire-8 (PHQ-8). The effect of MPVS  total and subscores on depression scores, quality of life subscores and quality of life total scores were examined using multiple linear regression analyses.

**Results:**

The majority of the patients reported that they had experienced at least one form of peer victimisation (95.8%, *n* = 229) during their school years. Higher levels of ‘verbal victimisation’, ‘attacks on property’ and higher total MPVS scores were significantly associated with lower social functioning; additionally, higher levels of ‘verbal victimisation’ were significantly associated with lower mental component summary scores in the quality of life assessment. Higher scores on all four subscales as well as higher total scores on the MPVS were significantly associated with more severe depressive symptoms.

**Conclusions:**

Given the high prevalence of peer victimisation in our sample and its associations with more severe depressive symptoms and lower quality of life, it is vital to implement interventions that prevent peer victimisation in educational and other social settings and to provide youth with strategies to more effectively manage instances of peer victimisation.

## Introduction

Peer victimisation or bullying is defined as the intentional and often recurrent infliction of physical or psychological harm on a person by peers who have a more dominant position in some regard to the person [1, 2]. Peer victimisation is common among adolescents. A report from the United Nations Educational, Scientific and Cultural Organisation (UNESCO) found that almost one in three (32%) children globally had been bullied in the preceding month [3]. However, the report also stated that there was a substantial regional variation in the prevalence of bullying across the world, with the proportion of students reporting that they had been bullied being highest in sub-Saharan Africa (48.2%) and lowest in Europe (25%), and Central America (22.8%). Research on peer-victimization in South-East Asian countries is relatively sparse. A review of South-East Asian countries found that the prevalence of bullying in the past month ranged from 29.6% in Indonesia (highest) to 15.7% in Myanmar (lowest). The Philippines and Thailand had prevalence rates of 24.3% and 17.5%, respectively, based on data from the Global School Health Survey (GSHS). In comparison, approximately 36% of Singapore and 43% of Thailand eighth-graders reported being bullied in the past month, based on data from the Trends in International Mathematics and Science Study (TIMMS) survey. The prevalence of bullying in both these countries was higher than the international average of 29% [4].

Peer victimization and bullying lead to negative consequences for adolescents, including poor academic performance [5], low self-esteem, loneliness [6], depression [7], self-harm, and suicide [8]. Peer victimisation especially when chronic in nature can be very stressful, which alone or by interacting with biological factors can influence the development of mental health problems [9]. Research has also linked an individual’s experiences of peer- victimisation with later psychological distress and lower levels of well-being. Using a longitudinal study design, Bowes et al. [10] examined the association between peer victimisation during early adolescence (13 years of age) and depression at 18 years of age. Compared with those who were not victimised, adolescents who reported being frequently victimised by peers had over a twofold increase in the odds of depression (based on the Clinical Interview Schedule-Revised) at 18 years of age even after adjusting for confounders.

Several factors have been found to be associated with peer victimisation. In particular, gender and age have been identified as indicators of the likelihood of adolescents being victimized at school. While some studies have indicated that males are more prone to peer victimisation [11, 12], others studies have suggested that there are no differences between male and female adolescents with respect to the frequency of experiencing peer victimisation [13]. In terms of age, the majority of studies have suggested that older students are less likely to experience peer-victimization than younger students [14, 15], possibly because older adolescents have greater awareness on how to stop harassment by peers [12]. Other studies have suggested that personal characteristics such as learning disabilities, poor academic performance, poor social skills, and emotional and behavioural problems also increase the likelihood of peer-victimization for adolescents [16–19]. Research has also suggested that the risk of being bullied is higher among adolescents with minority status, such as racial or ethnic minorities, sexual minority groups and youth with disabilities [20].

Singapore is a city-state in Southeast Asia comprising a multiethnic population. Studies on peer victimisation have been conducted largely in school-going adolescents. A study on students in primary and secondary schools in Singapore showed that 21% of primary and 25% of secondary students were victims of at least one form of bullying. Verbal bullying was most frequently reported, followed by relational, then physical, and cyberbullying was least frequent [21]. The students mentioned that bullying was associated with feelings of wanting to be alone, loss of appetite, problems in concentration, and sleeping at night. Another study by Ong and Elliot used a retrospective questionnaire to assess 400 young adults. Bullying was defined as ‘repeated and intentional attempts by others to hurt you or to cause distress to your daily life’. Nearly one quarter of the random sample of young adults stated that they had been bullied during their primary or secondary school days. However, the study did not find any gender differences in bullying [22].

Thus, while there are some studies, both globally and in Singapore on the prevalence and impact of peer-victimization in school populations, few studies have examined it among adolescent patients in a psychiatric setting. Additionally, we were not able to identify many studies that examined the association of peer victimisation and health related quality of life (HRQOL) among young people with mental illness. HRQOL is a multidimensional concept that includes physical, emotional, and social well- being and has thus emerged as an important measure of assessing the impact of health on overall functioning. Hence, the current study was designed to address these research gaps and to gain a deeper understanding of peer-victimization in a vulnerable population i.e., youth with mental illness.

The study was conducted as part of a larger study examining non-suicidal self-injury (NSSI) among patients in a tertiary psychiatric institution in Singapore [23]. This study aimed to examine the prevalence and correlates of recalled peer victimisation among youth with mental illness and to examine its association with depressive symptoms and HRQOL. We hypothesised that the prevalence of peer-victimization among patients would be high and that experiencing peer-victimisation would be associated with higher depressive symptoms and lower HRQOL after controlling for confounders.

## Methods

### Sample size

Since the primary aim of the larger study was to assess the prevalence of NSSI [23], the initial sample size calculation was based on the prevalence of NSSI in Singapore. However, since the primary aim of the current study is to determine the prevalence and correlates of peer victimisation among youth with mental illness, we calculated the sample size using a single proportion formula based on the prevalence of peer victimisation, which was reported to be 94.9% among children and youths with Autism Spectrum Disorder [24]. We found that we needed at least 207 subjects to achieve 3% precision of estimation at the 95% confidence interval. The number of participants who met the ‘youth’ criteria in the larger study was 239; hence, the sample size was assessed to be adequate for the current study.

### Participants

Participants were 239 respondents aged 15–24 years, where youth were defined according to the United Nation’s definition ‘as those persons between the ages of 15 and 24 years, without prejudice to other definitions by Member States’ which acknowledges that the meaning of the term ‘youth’ varies in different societies around the world [25]. Youths were recruited from the outpatient clinics of the Institute of Mental Health, Singapore (IMH). IMH is the only tertiary psychiatric institution in the city-state of Singapore that offers a range of psychiatric services for children, adolescents, adults, and elderly individuals.

### Procedure

Participants were recruited at the child and adult outpatient clinics in the IMH between October 2015 and June 2016. Ethics approval was attained from the National Healthcare Group Domain Specific Review Board, Singapore, and all participants gave written informed consent. Although a waiver of parental consent was obtained, the study was explained to parents who accompanied their children (i.e., those aged less than 21 years, as the age of the majority is 21 years in Singapore). Nevertheless, the research officers ensured that the self-administered questionnaires were completed independently by the participants. Participants were compensated with SGD 40 upon completion of the survey questionnaires, which took approximately 30 min.

### Assessments

Multidimensional Peer-Victimisation Scale [26]: This is a 16-item instrument that measures the extent to which young people were victimised by their peers. The items cover four aspects of peer victimisation: physical victimisation, social manipulation, verbal victimisation, and attacks on property (damaging, breaking or stealing property of victim), which are rated on a scale of 0 to 2 (0 = ‘Not at all’, 1 = ‘Once’, 2 = ‘More than once’). Scores on the total scale have a possible range of 0 to 32 and a possible range of 0 to 8 on each of the four subscales. The four subscales possess satisfactory internal consistency with Cronbach’s alpha coefficients of 0.85, 0.75, 0.77 and 0.73 for the physical victimisation, verbal victimisation, social manipulation and attacks on property subscales respectively. To our knowledge, the MPVS has not been used in Singapore. The Chinese version of the instrument has been used in China with good structural validity [27].

As this study primarily aimed to examine recalled experiences of peer victimisation, instructions were modified to encourage participants to think back to their experiences of peer victimisation in their life rather than their current experiences. This approach has been previously adopted by Cosgrove et al. [28].

Short Form-12 Survey (SF-12): This 12-item health-related quality of life measure assesses 8 domains related to quality of life. The response scales vary across items, with the number of response options ranging from 3 (physical functioning) to 6 (vitality and mental health) that are summarised into two summary scores, the Mental Component Summary (MCS) and Physical Component Summary (PCS) scores. The two scores range between 0 and 100, with increasing values equating to better health. The SF-12 has been found to be valid in the local population [29].

Patient Health Questionnaire Eight- Item (PHQ-8) [30]: This questionnaire asks if the patient had been bothered by symptoms of depression in the past 2 weeks. Participants are asked to rate how often each symptom occurred: 0 (not at all), 1 (several days), 2 (more than half the days), or 3 (nearly every day). The total score is determined by adding together the scores of each item. Total scores are rated as normal (0–2), mild (3–5), moderate (6–8), and severe (9–12) [30]. The PHQ-8 had excellent internal reliability in this sample (Cronbach’s alpha = 0.91).

Sociodemographic data were collected using a structured questionnaire that included information on age, gender, ethnicity, education, employment and marital status. The diagnosis of patients was extracted from their medical records.

## Statistical analysis

All statistical analyses were performed using SAS software version 9.4 [31] and MPLUS version 7.4. The mean and standard error were calculated for continuous variables, and frequencies and percentages were calculated for categorical variables. Confirmatory factor analysis was performed to establish the validity of the factor structure of the MPVS scale. Overall model fit was assessed using three goodness-of-fit indices: comparative fit index (CFI), Tucker‒Lewis index (TLI), and root mean square error of approximation (RMSEA). Hu and Bentler [32] suggested that cut-off values greater than 0.95 for TLI and CFI and less than 0.06 for RMSEA indicate a good fit, whereas RMSEA values ranging between 0.08 and 0.10 are considered mediocre fit [33]. Using multiple linear regression analysis, sociodemographic factors, i.e., age group, gender, ethnicity, marital status, level of education, employment status, and type of diagnosis, were entered as independent variables (IV) to predict MPVS subscores. The effect of MPVS subscores on PHQ-8 depression scores and SF-12 domains and total score (Model 1) were examined using multiple linear regression analyses after controlling for sociodemographic factors and type of diagnosis. Subsequently, the effect of MPVS subscores on quality of life domains was examined in the final model using multiple linear regression analyses after controlling for sociodemographic factors, type of diagnosis and PHQ-8 depression score. Statistical significance was set at a p value of less than 0.05.

## Results

### Descriptive statistics

The mean age of the participants was 19.4 (standard deviation; SD: 2.6) years, with the majority being Chinese (*n* = 182, 76.2%), and single (*n* = 236, 98.7%). There were slightly more males than females (52.7% versus 47.3%). Most participants were diagnosed with mood disorder (37.4%), followed by adjustment disorder (20.6%) and anxiety disorder (17.2%) (Table 1).

**Table 1:** Socio-demographic characteristics of the sample

Variables	Total sample (n = 239)	
	Mean	SD
**Age** (range 15-24y)	19.4	2.6
	n	%
**Gender**		
Female	113	47.3
Male	126	52.7
**Ethnicity**		
Chinese	182	76.2
Malay	39	16.3
Indian	11	4.6
Others	7	2.9
**Marital Status**		
Single	236	98.8
Married	1	0.4
Separated	1	0.4
Divorced	1	0.4
**Education**		
Primary	19	7.9
Secondary	28	11.7
‘O’ / ‘N’ level	82	34.3
‘A’ level	20	8.4
Vocational Training	25	10.5
Diploma and Degree	65	27.2
Employment*		
Employed	37	15.4
Student (full-time)	117	50.0
National Service	63	26.3
Unemployed	15	6.2
Diagnosis*		
Adjustment disorders	49	20.5
Anxiety disorders	41	17.2
Behavioural and developmental disorders	17	7.1
Mood disorders	89	37.2
Others	17	7.1
Schizophrenia spectrum	26	10.9

^*^Data on employment from 7 participants were missing

A level—certificate given to those who have completed junior college which is equivalent to high school

O’/’N’ level—secondary school leaving certificate examination

The majority of the patients reported that they had experienced at least one form of peer victimisation (95.8%, *n* = 229) over their years of schooling. Patients reported that they had been victimized by their peers ‘once’ or ‘more than once’ during their school years with ‘called me names’ (85.4%), ‘made fun of me for some reason (83.7%), and ‘refused talk to me’ (73.6%) being most frequently endorsed. The mean total score of the MPVS scale was 16.1 (9.3).

The four-factor structure of the MPVS scale proposed by Mynard and Joseph [26], was examined using confirmatory factor analysis. We found that the fit indices were good (χ^2^ (df) = 267.848(98), CFI = 0.99, TLI = 0.99, RMSEA = 0.07), as each item had a high factor loading within the dimension (results are available upon request). The Cronbach’s alpha for the four factors ‘physical victimisation’, ‘social manipulation’, ‘verbal victimisation’ and ‘attacks on property’ were 0.90, 0.85, 0.82 and 0.83 respectively. The mean scores of physical victimisation, social manipulation, verbal victimisation, and attacks on property were 2.22 (2.82), 4.85 (2.90), 5.59 (2.70) and 3.45 (2.85), respectively.

### Sociodemographic and clinical correlates of the MPVS subscales

Multiple linear regression analyses showed that gender was significantly associated with scores on the MPVS subscales and the total score. Male gender was associated with a higher likelihood of ‘physical victimisation’, ‘attacks on property’ and total scores on the MPVS. Higher levels of education, i.e., ‘A’ level (equivalent to high school) versus those with lower education, i.e., ‘O’/’N’ level (secondary school completion), were associated with lower odds of ‘social manipulation’, while those currently pursuing secondary education or who had not passed the secondary school examination had higher odds of reporting ‘attacks on property’ compared to someone who had completed secondary school. However, these findings were not consistent across all levels of education (Table 2).

**Table 2:** Sociodemographic and clinical correlates of the multidimensional peer victimisation subscales

	Physical victimisation			Social manipulation			Verbal victimisation			Attacks on property			Total scores		
	B	95% CI	p	B	95% CI	p	B	95% CI	p	B	95% CI	p	B	95% CI	p
Age	0.09	− 0.10–0.29	0.346	− 0.09	− 0.30–0.12	0.403	− 0.04	− 0.24–0.16	0.720	0.17	− 0.04–0.37	0.109	0.13	− 0.53–0.80	0.692
Sex															
Female	Ref														
Male	1.83	0.89–2.78	< 0.001	0.11	− 0.90–1.12	0.832	0.93	− 0.03–1.90	0.058	1.07	0.09–2.05	0.032	3.95	0.72–7.18	0.017
Ethnicity															
Chinese	Ref														
Malay	0.44	− 0.57–1.46	0.386	− 0.02	− 1.10–1.06	0.969	0.02	− 1.01–1.06	0.965	− 0.19	− 1.24–0.86	0.727	0.26	− 3.20–3.72	0.882
Indian	− 0.03	− 1.71–1.64	0.968	− 0.48	− 2.27–1.32	0.601	− 1.00	− 2.72–0.72	0.252	− 1.01	− 2.75–0.74	0.257	− 2.52	− 8.26–3.22	0.388
Others	1.71	− 0.56–3.99	0.140	0.75	− 1.69–3.18	0.547	0.32	− 2.01–2.65	0.789	1.48	− 0.88–3.85	0.218	4.26	− 3.53–12.05	0.283
Education															
***‘O’/‘N’ level***	Ref														
***Primary***	1.04	− 0.46–2.53	0.174	− 0.13	− 1.73–1.47	0.877	− 0.22	− 1.75–1.32	0.781	0.50	− 1.06–2.06	0.527	1.19	− 3.93–6.32	0.647
***Secondary***	0.88	− 0.41–2.16	0.180	0.24	− 1.14–1.61	0.734	− 0.02	− 1.34–1.30	0.974	1.51	0.18–2.85	0.027	2.61	− 1.80–7.01	0.244
***‘A’ level***	− 0.06	− 1.43–1.31	0.928	− 1.59	− 3.05 to − 0.12	0.034	− 0.26	− 1.67–1.14	0.713	− 0.87	− 2.30–0.55	0.228	− 2.79	− 7.48–1.91	0.243
***Vocational***	1.20	− 0.09–2.50	0.069	0.08	− 1.31–1.47	0.914	0.64	− 0.69–1.98	0.341	0.67	− 0.68–2.03	0.327	2.60	− 1.85–7.05	0.251
***Any Diploma & Degree***	0.76	− 0.26–1.78	0.145	0.64	− 0.45–1.74	0.246	0.76	− 0.29–1.80	0.156	0.46	− 0.60–1.52	0.393	2.62	− 0.88–6.11	0.141
Employment															
***Student (full-time)***	***Ref***														
Employed	− 0.64	− 1.73–0.45	0.248	0.04	− 1.13–1.21	0.947	0.15	− 0.96–1.27	0.787	− 0.40	− 1.53–0.74	0.489	− 0.85	− 4.58–2.89	0.655
National Service	− 0.74	− 1.89–0.41	0.207	− 0.39	− 1.62–0.84	0.534	− 0.79	− 1.97–0.39	0.188	− 0.67	− 1.87–0.52	0.267	− 2.59	− 6.52–1.34	0.196
Unemployed	0.17	− 1.43–1.77	0.832	− 0.22	− 1.93–1.49	0.803	− 0.16	− 1.80–1.48	0.852	0.42	− 1.25–2.08	0.621	0.22	− 5.26–5.70	0.937
Diagnosis															
Mood disorder	***Ref***														
Schizophrenia spectrum	− 0.31	− 1.59–0.98	0.637	− 1.57	− 2.94 to − 0.19	0.026	− 1.13	− 2.45–0.18	0.091	− 1.63	− 2.97 to − 0.29	0.017	− 4.64	− 9.04–− 0.24	0.039
Anxiety disorder	− 0.21	− 1.27–0.86	0.702	− 1.05	− 2.19–0.09	0.070	− 0.35	− 1.44–0.74	0.524	− 0.99	− 2.10–0.11	0.078	− 2.60	− 6.23–1.03	0.160
Others	0.40	− 1.18–1.98	0.617	− 0.61	− 2.30–1.08	0.481	− 0.98	− 2.60–0.64	0.234	− 0.13	− 1.77–1.51	0.877	− 1.32	− 6.73–4.10	0.632
Adjustment disorders	− 0.01	− 1.05–1.04	0.991	− 0.70	− 1.82–0.43	0.223	− 0.05	− 1.13–1.02	0.922	− 0.44	− 1.53–0.65	0.428	− 1.19	− 4.78–2.39	0.512
Behavioural and developmental disorders	− 1.17	− 2.67–0.34	0.129	− 2.26	− 3.87 to − 0.65	0.006	− 0.68	− 2.23–0.87	0.387	− 0.86	− 2.34–0.63	0.257	− 4.91	− 10.08–0.25	0.062

A level—certificate given to those who have completed junior college which is equivalent to high school

O’/N’ level (secondary school leaving certificate examination

Those diagnosed with schizophrenia spectrum disorder, behavioural and developmental disorder (vs. mood disorders) were significantly associated with a lower likelihood of ‘social manipulation’, while the presence of schizophrenia spectrum disorder was associated with a lower likelihood of ‘attacks on property’ compared to mood disorders. Those with schizophrenia spectrum disorder were associated with lower total scores than those with mood disorders (Table 2).

### Relationship between MPVS subscales, quality of life domains and PHQ-8 depression symptoms

After controlling for sociodemographic factors and type of diagnosis, higher scores on ‘social manipulation’, ‘attacks on property’ and total scores on the MVPS were significantly associated with lower scores on bodily pain, vitality, social functioning, role-emotional, mental health and mental component summary scores on the SF-12. Higher scores on ‘verbal victimisation were significantly associated with lower social functioning, role-emotional, mental health and mental component summary scores on the SF-12. Higher ‘physical victimisation’ scores were significantly associated with lower role-physical scores (data available on request).

After controlling for sociodemographic factors, type of diagnosis and PHQ-8 total scores in multiple linear regression analysis (final model), we found that higher ‘verbal victimisation’, ‘attacks on property’ and total MPVS scores were significantly associated with lower social functioning scores on the SF-12; additionally, higher ‘verbal victimisation’ scores were significantly associated with lower mental component summary scores on the SF-12 (Table 3). After controlling for sociodemographic factors and type of diagnosis, higher scores on all four subscales as well as total score on MPVS were significantly associated with higher depressive symptoms as measured by the PHQ-8 (Table 3).

**Table 3:** Relationship between MPVS subscales, quality of life domains and PHQ-8 depression symptoms

	Physical victimisation			Social manipulation			Verbal victimisation			Attacks on property			Total scores		
	B	95% CI	*p*	B	95% CI	*p*	B	95% CI	*p*	B	95% CI	*p*	B	95% CI	*p*
SF-12*															
Physical functioning	0.12	− 0.35–0.59	0.629	0.27	− 0.17–0.71	0.235	0.30	− 0.16–0.76	0.196	− 0.08	− 0.54–0.38	0.736	0.06	− 0.08–0.20	0.419
Role-physical	− 0.30	− 0.78–0.18	0.218	− 0.07	− 0.52–0.38	0.761	− 0.16	− 0.63–0.31	0.495	− 0.20	− 0.67–0.27	0.402	− 0.07	− 0.21–0.08	0.355
Bodily pain	0.20	− 0.31–0.71	0.435	− 0.30	− 0.78–0.18	0.215	− 0.24	− 0.74–0.26	0.350	− 0.44	− 0.94–0.05	0.079	− 0.08	− 0.23–0.08	0.328
General health	0.19	− 0.34–0.72	0.479	0.02	− 0.47–0.52	0.929	0.06	− 0.45–0.58	0.813	− 0.15	− 0.66–0.36	0.565	0.01	− 0.15–0.17	0.892
Vitality	0.03	− 0.46–0.52	0.907	− 0.26	− 0.72–0.20	0.270	0.03	− 0.45–0.51	0.906	− 0.32	− 0.80–0.15	0.185	− 0.05	− 0.20–0.09	0.495
Social functioning	− 0.05	− 0.53–0.43	0.843	− 0.43	− 0.88–0.02	0.061	− 0.64	− 1.11 to − 0.18	0.007	− 0.77	− 1.23 to − 0.31	0.001	− 0.18	− 0.32–− 0.04	0.014
Role-emotional	0.001	− 0.48–0.48	0.997	− 0.04	− 0.50–0.41	0.845	− 0.36	− 0.83–0.11	0.131	− 0.17	− 0.64–0.30	0.475	− 0.05	− 0.20–0.09	0.461
Mental health	0.39	− 0.01–0.78	0.054	− 0.20	− 0.58–0.17	0.284	− 0.22	− 0.61–0.17	0.262	0.03	− 0.36–0.42	0.866	0.00	− 0.12–0.12	0.956
Physical component summary	− 0.03	− 0.47–0.41	0.895	0.05	− 0.37–0.46	0.820	0.13	− 0.30–0.57	0.538	− 0.26	− 0.69–0.17	0.232	− 0.01	− 0.14–0.12	0.885
Mental component summary	0.17	− 0.24–0.58	0.413	− 0.32	− 0.70–0.07	0.103	− 0.46	− 0.85 to − 0.06	0.024	− 0.25	− 0.65–0.15	0.214	− 0.08	− 0.20–0.04	0.184
PHQ-8 total score **	0.45	0.14–0.76	0.005	0.50	0.21–0.78	0.001	0.50	0.19–0.80	0.001	0.58	0.29–0.88	< 0.01	0.19	0.10–0.27	< 0.001

^*^Regression analysis controlled for socio-demographic factors, diagnostic groups and PHQ-8 score

^**^Regression analysis controlled for socio-demographic factors and diagnostic groups

## Discussion

While peer victimisation or bullying is a common problem worldwide, the prevalence of 95.8% in our sample is extremely high. Other studies that have used a similar methodology i.e., examined peer victimisation at any point in the lifetime of the adolescent or young adult from the school or general population have reported a much lower prevalence. Mynard and Joseph used the MPVS in a secondary school sample, comprising children aged 11–16 years, and found that 43% of students had been bullied at some point [26]. A study in Singapore that examined young adults’ recall of peer victimisation during their primary or secondary school days and found that 25.3% of the youth had experienced bullying [22]. However, the prevalence of peer-victimization in youths and adolescents with disabilities and autism spectrum disorder (ASD) was higher and comparable to our results. McNicholas et al. [33] conducted a retrospective study among college students with disabilities and examined their recollection of peer victimisation during middle school and high school years; the results indicate that two-thirds of the participants had experienced peer victimisation. A study among a French population of children and youths with ASD found that 94.9% of the subjects had been victimized during their lifetime [24].

The prevalence of bullying in the current sample could be high because youth who have mental illness may have poor social skills, appear vulnerable and find it more difficult to fit in and make friends, thereby rendering them an easy target for peer-victimization. On the other hand, peer-victimization can also be a significant risk factor for psychological distress. A reciprocal relationship between internalizing symptoms and relational victimisation was reported by McLaughlin [35] however Bond et al. [36] found that while victimisation was predictive of depression and anxiety in girls, prior mental health problems were not predictive of victimisation. On the other hand, a longitudinal study found that depression predicted victimisation among girls, while victimisation predicted depression among boys [37].

Verbal victimisation in the form of ‘called me names’ and ‘made fun of me for some reason’ and relational victimisation in the form of ‘refused talk to me’ were the most frequently endorsed forms of peer victimisation reported by our respondents. Weiner and Mak [38] and Koh and Tan [21] similarly reported that verbal victimisation was the most frequent, while relational and physical victimisation were less frequent. Hosozawa et al. [39] examined the prevalence of victimisation in 15-year-olds across 71 countries. Students reported frequencies of relational, physical, and verbal victimisation during the last 12 months. They similarly found that verbal and relational victimisation were more frequent than physical victimisation over the past 12 months.

In our study, male gender was found to be significantly associated with ‘physical victimisation’, ‘attacks on property’ and the total scores on the MPVS. Gender differences have been observed in several studies in terms of the type of victimisation. Mynard and Joseph [26] reported that boys had higher mean scores on both physical victimisation and attacks on property. Their study also found that girls scored higher than boys in the domain of social manipulation which was not found in the current study. Cross-cultural data suggest that physical aggression is more common among boys [40, 41]. Previous research has linked aggressive behaviour to testosterone, which increases during puberty with levels remaining elevated in young male adults [42].

Our study found that multiple domains of quality of life, bodily pain, vitality, social functioning, role-emotional and role- physical domains of the SF-12 were adversely affected by peer victimisation. Interestingly, domains related to physical health were not significantly affected by peer victimisation in this sample. The mental health component score was also significantly lower among those with various forms of peer victimisation. However, on controlling for PHQ-8 scores, we found that higher ‘verbal victimisation’ and ‘attacks on property’ and total MPVS scores were significantly associated with lower social functioning scores; we are unable to explain why these two forms of peer-victimization were significantly associated with lower social functioning.

These results show that peer-victimization affects the quality of life of these young people above and beyond the severity of depression experienced by them. Few studies have examined the relation between peer victimisation and quality of life. Peer victimisation was negatively associated with total scores on the Paediatric Quality of Life Inventory Child Versions and scores on the psychosocial subscale among adolescents with acne vulgaris [43]. Other studies in paediatric populations have similarly reported that victimisation was found to predict poor quality of life [44, 45].

Higher scores in all four domains of the MPVS were associated with more severe depression as measured by the PHQ-8. Many studies have reported that those experiencing peer victimization have more depressive symptoms than those who do not [46, 47]. Klomek et al. [48] proposed a “self-concept perspective” which suggests that peer victimisation undermines an adolescent’s self-concept thus triggering depression and related constructs. Other studies have suggested that those with anxiety and depression may be more likely to perceive some of their experiences as victimisation compared to those without these internalising disorders [49].

Limitations of the current study include the use of self-report data and the cross-sectional design of our study. The use of multiple sources of information such as including parents and teachers to determine victimisation would have strengthened our findings. However, victimisation is an intensely personal experience, and teachers and parents may not always be aware of it. The cross-sectional nature of our study precludes discussion of temporal relationships between peer victimisation, severity of depression and quality of life. The sample being a convenient sample, may not be representative of youths seeking treatment in the tertiary psychiatric institution or youths with mental illness in general thus limiting the generalisability of our results. The MPVS does not include cyber bullying which is emerging as a significant component of victimisation in youth given their extensive use of social media, and thus the true extent of peer victimisation may have been under-estimated by the current study. Lastly, the social changes imposed by the Covid-19 pandemic may have changed the prevalence and impact of peer-victimisation.

Our findings have several implications. Given the high prevalence of peer victimisation in our sample associated with severity of depressive symptoms and lower quality of life, it is extremely important to incorporate interventions that provide youth with strategies to manage future instances of peer victimisation more effectively. Educational institutes need to be aware of the extent of peer victimisation and its ramifications. The school must provide a safe environment by establishing clear rules, supervision in places where bullying is likely to occur, increasing parental involvement when incidents occur and playing a supportive role by providing counselling to both the bullies and the victims. Thus, schools can holistically prevent peer-victimization in the school setting and by educating and counselling the youth to prevent the behaviour outside the school settings as well. Such multidisciplinary whole school approaches that comprise a combination of schoolwide disciplinary measures, teacher training, curriculum interventions, conflict resolution training, and individual counselling have shown positive results [50]. Studies have found that resilience is an important protective factor, and that it both prevents bullying and mitigates its effect [51]. Thus, brief growth mindset interventions that build resilience, i.e., interventions that promote positive adaptation despite adversity would reduce the behavioural and emotional symptoms associated with victimization [52]. However, there is a need to both develop such interventions that would be appealing to adolescents and monitor their outcomes.

The current study is among the first to report on peer-victimization among a psychiatric outpatient sample. The study found a high prevalence of peer victimisation among youth seeking treatment at a tertiary psychiatric institution that was associated with severity of depression and lower quality of life. Longitudinal studies are needed both in community and patient samples to understand the phenomenon in further depth. Mediational analyses to understand the link between peer victimisation and mediators including self-esteem, personality, classmate support, resilience and outcomes in youth are needed. Last, the study calls for evidence-based interventions to mitigate peer victimisation which must subsequently be evaluated from the perspectives of both the victimised youth and those doing the victimisation.

## Data Availability

The datasets used and/or analysed during the current study are available from the corresponding author on reasonable request.
